# Modulation of Cholesterol Pathways in Human Macrophages Infected by Clinical Isolates of *Leishmania infantum*

**DOI:** 10.3389/fcimb.2022.878711

**Published:** 2022-04-29

**Authors:** José Ignacio Manzano, Ana Perea-Martínez, Raquel García-Hernández, Eduardo Andrés-León, Laura C. Terrón-Camero, José Antonio Poveda, Francisco Gamarro

**Affiliations:** ^1^ Instituto de Parasitología y Biomedicina “López-Neyra”, Consejo Superior de Investigaciones Científicas (IPBLN-CSIC), Parque Tecnológico de Ciencias de la Salud, Armilla, Spain; ^2^ Instituto de Investigación, Desarrollo e Innovación en Biotecnología Sanitaria de Elche (IDiBE), and Instituto de Biología Molecular y Celular (IBMC), Universidad Miguel Hernández, Elche, Spain

**Keywords:** human macrophages, *Leishmania infantum*, host-cell modulation, plasma membrane fluidity, host cholesterol content, transcriptomic analysis

## Abstract

To increase our understanding of factors contributing to therapeutic failure (TF) in leishmaniasis, we have studied some plasma membrane features of host THP-1 cells infected with clinical isolates of *Leishmania infantum* from patients with leishmaniasis and TF. The fluorescent probes DPH and TMA-DPH were used to measure changes in membrane fluidity at various depths of the plasma membranes. Steady-state fluorescence anisotropy of DPH embedded in the infected THP-1 membranes showed a significant increase, thereby suggesting a substantial decrease in plasma membrane fluidity relative to controls. Considering that cholesterol affects membrane fluidity and permeability, we determined the cholesterol content in plasma membrane fractions of human macrophages infected with these *L. infantum* lines and observed a significant increase in cholesterol content that correlates with the measured decrease in plasma membrane fluidity. In order to define the pathways that could explain the increase in cholesterol content, we studied the transcriptomics of the cholesterol-enriched pathways in host THP-1 cells infected with TF clinical isolates by RNA-seq. Specifically, we focused on four enriched Gene Ontology (GO) terms namely cholesterol efflux, cholesterol transport, cholesterol metabolic process and cholesterol storage. Additionally, we analyzed the genes involved in these pathways. Overall, this study shows that these clinical isolates are able to modulate the expression of specific genes in host cells, thereby modifying the cholesterol content in plasma membranes and inducing changes in plasma membrane fluidity that could be associated with the parasite’s ability to survive in the host macrophages, thereby possibly contributing to immune evasion and TF.

## Introduction

The ability of intracellular pathogens to modulate host cells in order to survive and to evade the host immune response has been described (Chaussabel et al., 2003; Zhang et al., 2010; Ramírez et al., 2012). Indeed, this strategy could contribute to therapeutic failure (TF) in patients infected with these pathogens. One of the host cell factors targeted is cholesterol, a major lipid component of eukaryotic membranes that plays a role in signal transduction (Incardona and Eaton, 2000), maintenance of membrane fluidity (Shinitzky and Inbar, 1974) and transport (Bastiaanse et al., 1997), and is also relevant for the intracellular survival and replication of pathogens (Portugal et al., 2008; Ehrenman et al., 2013; Johndrow et al., 2014). Modulation of host cholesterol by intracellular pathogens may result in impairment of the host’s immune response for their own benefit (Rub et al., 2009; Roy et al., 2016).

Structurally, cholesterol affects membrane fluidity and permeability, with higher cholesterol levels increasing membrane rigidity (Gennis, 1989). Membrane fluidity plays an important role in cellular functions since the activity of membrane proteins is modulated by the surrounding lipid environment. Furthermore, alteration of cholesterol pathways may also affect micro-domain structure and organization of cell membranes, thereby inducing changes in cell signaling (Ibarguren et al., 2014). Due to the important biological roles of cholesterol, its cellular levels are precisely controlled (Simons and Ikonen, 2000; Soccio and Breslow, 2004; Maxfield and Tabas, 2005; Ikonen and Jansen, 2008) by way of a regulatory feedback circuit that senses the levels of cholesterol in cell membranes and modulates the transcription of genes encoding the enzymes for cholesterol biosynthesis and uptake (Lange et al., 1999; Radhakrishnan and McConnell, 2000).

One of the most relevant parasitic diseases is leishmaniasis, a neglected tropical disease caused by the protozoan parasite *Leishmania* which, in its visceral form, is lethal if left untreated. Several studies suggest that *Leishmania* infection induces changes in the transcriptome of infected host cells (García-Hernández et al., 2022; Perea-Martínez et al., 2022). In addition, transcriptomic studies of infected host cells have shown that *Leishmania* infection modulates the expression of genes associated with cholesterol biosynthesis, uptake, and efflux, which might play a role in the internalization and survival of parasites (Osorio y Fortéa et al., 2009; Rabhi et al., 2012; Lecoeur et al., 2013) by influencing the host cellular signaling (Rub et al., 2009). However, contradictory reports about the status of host cellular cholesterol content after *Leishmania* infection have been published (Osorio y Fortéa et al., 2009; Rabhi et al., 2012; Lecoeur et al., 2013), with some studies describing an increased cholesterol content in infected macrophages (Osorio y Fortéa et al., 2009; Rabhi et al., 2012; Mukherjee et al., 2014), whereas others have reported that *Leishmania* induces cholesterol depletion in the infected host cells (Chakraborty et al., 2005). Similarly, other protozoan parasites, such as *Toxoplasma gondii*, induce a reduction in host plasma membrane cholesterol content that inhibits parasite entry (Coppens and Joiner, 2003), thereby affecting lipid raft-dependent processes (Chakraborty et al., 2005; Mukherjee et al., 2014).

As relevant intracellular pathogens, *Leishmania* parasites have developed strategies to evade host immune mechanisms in order to survive. Thus, some authors have described that infection with *Leishmania* parasites modifies membrane cholesterol content, thereby inducing alteration of the immune response required to control the progression and evolution of this parasitic infection, thus contributing to immune evasion of the parasites (Rub et al., 2009; Roy et al., 2016).

Herein we study the plasma membrane fluidity and cholesterol content in plasma membrane fractions of THP-1 cells infected with clinical isolates of *Leishmania infantum* from patients with leishmaniasis and TF. Additionally, we study the transcriptomic analysis of sterol and cholesterol pathways of *L. infantum*-infected host THP-1 cells at a later time point of infection by RNA-seq. Our findings show that various important cholesterol genes and pathways are modulated by these parasites and could therefore be associated with the parasite’s ability to survive in the host macrophages, thus leading to immune evasion and probably contributing to TF.

## Materials and Methods

### Chemical Compounds

Triton X-100, 4’,6-diamidino-2-phenylindole dilactate (DAPI), phorbol 12-myristate 13-acetate (PMA), 1,6-Diphenyl-1,3,5-hexatriene (DPH), 1-[4-(trimethylamino) phenyl]-6-phenyl-1,3,5-hexatriene (TMA-DPH), Phenylmethylsulfonyl Fluoride (PMSF), NaCl, HEPES, EDTA, TRIS and sodium dodecyl sulfate (SDS) were purchased from Sigma-Aldrich (St. Louis, MO). L-glutamine and penicillin/streptomycin were obtained from Gibco. Bradford reagent was purchased by Bio-Rad (Hercules, USA). Protease inhibitors were provided by Thermo Scientific (Waltham, USA). All chemicals were of the highest quality available.

### Culture of *L. infantum* Lines and THP-1 Cells

We used promastigotes of *L. infantum* lines: (i) JPCM5 (MCAN/ES/98/LLM-877) as a reference control line; and (ii) LLM2070, LLM2165, LLM2255 and LLM2221 lines as *L. infantum* lines isolated from HIV positive patients with visceral leishmaniasis and TF, treated with liposomal amphotericin B and antiretroviral therapy, from the WHO Collaborating Center for Leishmaniasis, Instituto de Salud Carlos III (ISCIII) (Dr. F. Javier Moreno). The sensitivity profile of the *L. infantum* lines has been previously described (Perea-Martinez et al., 2022). All these *L. infantum* lines were grown at 28°C in RPMI 1640-modified medium (Invitrogen) supplemented with 10% hiFBS (Invitrogen), as described (Manzano et al., 2019). Human myelomonocytic THP-1 cells were grown at 37°C and 5% CO_2_ in RPMI-1640 medium supplemented with 10% iFBS, 2 mM glutamate, 100 U/mL penicillin and 100 mg/mL streptomycin as described (Sanchez-Fernandez et al., 2019).

### *In Vitro* Macrophage Infection

THP-1 cells (3x10^7^ cells in 175-cm^2^ flasks or 5x10^5^ cells/well in 24-well plates) were differentiated to macrophages with 20 ng/mL of PMA treatment for 48 h followed by 24 h of culture in fresh medium. For *in vitro* infection, macrophage-differentiated THP-1 cells were infected with different *L. infantum* stationary-phase promastigotes incubated for 72 h in acid medium plus 10% hiFBS or heat-killed promastigotes (incubated for 1 h at 65°C) at a macrophage/parasite ratio of 1:10 (Perea-Martínez et al., 2022). Infected macrophages were incubated in RPMI 1640 medium plus 10% hiFBS at 37°C and 5% CO_2_ for 96 h. On the other hand, to determine the percentage of infection and the average of amastigotes by cell, macrophages were infected with the same *L. infantum* lines and treated in parallel with identical conditions described above. For microscopy visualization, cells were fixed for 30 min at 4°C with 2.5% paraformaldehyde in PBS and permeabilized with 0.1% Triton X-100 in PBS for 30 min. The infection level (60-80%) and mean number of intracellular parasites/cell (7-10) were detected by nuclear staining with DAPI (Invitrogen) (**Supplementary Table 1**). Additionally, we used macrophages that had been allowed to phagocytose heat-killed parasites and uninfected macrophages as controls. In this way, we will identify changes in gene expression that represent a general result of phagocytosis and phagolysosome formation rather than being specific to *Leishmania* infection. The abbreviations for different infected host cell lines used are the following: (i) Hi-LJPC for THP-1 cells infected with *L. infantum* JPCM5 line; (ii) Hi-L2070 for THP-1 cells infected with *L. infantum* LLM2070 line; (iii) Hi-L2165 for THP-1 cells infected with *L. infantum* LLM2165 line; (iv) Hi-L2255 for THP-1 cells infected with *L. infantum* LLM2255 line; and (v) Hi-L2221 for THP-1 cells infected with *L. infantum* LLM2221 line.

### Preparation of Infected THP-1 Cells Surface Membrane Fraction

Approximately 3x10^7^ *L. infantum* infected THP-1 cells cultured in 175-cm^2^ flasks were washed three times with PBS and subsequently detached and broken with cold hypotonic buffer (10 mM Tris-HCl pH 7.4, 2 mM EDTA), a mixture of protease inhibitors (Thermo Scientific) and PMSF (Sigma-Aldrich). The cells were centrifuged at 12000 g for 20 min to eliminate unbroken cells and amastigotes. Then, the supernatant was collected and centrifuged at 50000 g for 60 min to discard the rest of the cellular components. The pellet containing the *Leishmania* depleted THP-1 plasma membrane fractions was resuspended in a buffer containing 20 mM HEPES pH 7.4 and 150 mM NaCl enriched with protease inhibitors. Protein content was quantified using Bradford assay (Bio-Rad).

### SDS-PAGE and Western Blotting

In order to discard the presence of parasites in the THP-1 plasma membrane samples, and consequent validation of the technique, the absence of *Leishmania* membranes from the isolated fractions was checked. For that purpose, we analyzed the presence of the *Leishmania* surface protease GP63 in the samples. Additionally, we detected Na^+^/K^+^ ATPase, an enzyme located in the plasma membrane of cells to confirm the isolation of the infected THP-1 membrane fraction (**Figure S1**).

Consequently, protein samples were fractionated by SDS-PAGE under standard conditions and electrotransferred onto Immobilon-P membranes (Millipore). Subsequently, membranes were cut in order to perform incubations with different antibodies on the same loaded samples. Immunodetection was performed by using a 1:5000 dilution of mouse monoclonal anti-(GP63) (Life Span BioSciences) or mouse monoclonal anti-(Na^+^/K^+^ ATPase, α1 subunit) (Sigma-Aldrich) in phosphate-buffered saline (PBS) plus 0.01% Tween 20 and 0.1% bovine serum albumin (BSA). After washing, membranes were incubated with horseradish peroxidase conjugated secondary goat anti-mouse immunoglobulin G (DAKO) using a 1:5000 dilution. Signal was detected employing the ECL chemiluminescent substrate (Pierce).

### Cholesterol Content Measurement

Cholesterol content in the plasma membrane fractions of THP-1 cells was measured using Amplex Red Cholesterol Assay kit (Life Technologies, Oregon, USA), following the manufacturer’s protocol. For each reaction, we used 2.5 μg protein of plasma membrane that correspond with the fraction obtained after depleting *Leishmania* amastigotes following the protocol described above. Briefly, reactions took place in a 96-well plate by the addition of 50 μL of assay sample and 50 μL of Amplex Red working solution. The 5 mL of Amplex Red working solution were prepared previously, containing Amplex Red reagent, horseradish peroxidase, cholesterol oxidase and cholesterol esterase in the indicated amounts in the kit. The reactions were incubated for 30 min at 37°C protected from light. Finally, the fluorescence intensity was measured at 540 nm (excitation)/590 nm (emission) using an Infinite F200 microplate reader (Tecan Austria GmbH, Austria). The cholesterol contents were determined using 0-6 µg/mL cholesterol standards (provided in the Amplex Red kit and used as reference to obtain the standard curves).

### Membrane Fluidity Studies Evaluated by Fluorescence Anisotropy

The changes in membrane fluidity of THP-1 cells infected with different clinical *L. infantum* isolates were measured by assessing fluorescence depolarization of the probes DPH and TMA-DPH as described (Manzano et al., 2011). Briefly, *L. infantum-* infected THP-1 plasma membrane samples that correspond with the fractions obtained after depleting *Leishmania* amastigotes following the protocol of preparation described above (0.14–0.15 mg/mL final protein concentration) in 10 mM HEPES, 145 mM NaCl, pH 7.4, buffer were incubated with DPH or TMA-DPH probes in *N*,*N*′-dimethylformamide (DMF) in the dark for 30 min at room temperature at a 1/2500 probe/protein weight ratio. The final DMF concentration in the membrane suspension was always < 0.05%. A Cary Eclipse spectrofluorometer (Agilent Technologies) was used to measure the steady-state anisotropy (r), quantifying the vertical and horizontal components of the fluorescence emission with excitation polarized vertically. The grating factor (GF) is specific for the instrument and is defined by the ratio of the fluorescence intensities with polarizers in the horizontal and vertical position (excitation and emission respectively), and both horizontal, respectively. The slit widths for both excitation and emission were 5 nm and the integration time was 1 s. The excitation wavelength for DPH and TMA-DPH was 360 nm, with emission being monitored at 430 nm. The data for each experiment were calculated as the average of 10-s anisotropy measurements at a fixed temperature of 37°C due to its physiological relevance.

### Transcriptomic Data Analysis

The sequences of THP-1 cells infected with TF lines employed in the present manuscript were obtained from a previous RNA-seq project available at NCBI Short Read Archive (SRA) under the accession number: PRJNA781438, as described (Perea-Martínez et al., 2022). Briefly, miARma-Seq pipeline was used (Andrés-León et al., 2016) to perform sample quality control, the alignment of samples and the calculation of differentially expressed genes. Therefore, sequences were aligned by using HISAT2 (Kim et al., 2015), against the protein coding genes from the *Homo sapiens* Gencode version GRCh38-M26 genome-build. Gene expression values were used to compare the three replicates of THP-1 infected with *L. infantum* lines adjusted by control samples (uninfected cells and phagocytosis control) using edgeR (Nikolayeva and Robinson, 2014; Ritchie et al., 2015). All genes having a false discovery rate (FDR) value < 0.05 were included in the study. Log_2_FC was used to evaluate the significance and the change in expression of a gene respectively between different types of samples. We considered a gene to be differentially expressed (DEGs) using the parameters Log_2_FC value ≥ 0.58 or ≤ -0.58 and FDR value ≤ 0.05.

### Enrichment Analysis

The cluster Profiler Bioconductor package (Yu et al., 2012) was used with the aim of identifying differential gene expression effects by carrying out a functional enrichment study. For this purpose, all expressed genes of the RNA-seq assay were compared obtaining the Gene Ontology (GO) terms from the Bioconductor *Homo sapiens* database and associated to Entrez gene identifiers in an *orgDB* R object through the *AnnotationForge* package to be used with *clusterProfiler*. Significant (*p* value ≤ 0.05) terms related to sterol pathways from the GO: biological process (BP) ontology were selected and the complete ancestor chart of the BP ontology (Binns et al., 2009) was created with QuickGO tool. The genes from the GO terms: cholesterol efflux (GO:0033344), cholesterol transport (GO:0030301), cholesterol storage (GO:0010878) and cholesterol metabolic process (GO:0008203) were selected, and heatmaps were created with R 4.2.1 program (Galili et al., 2018) using the heatmaply package (Team, 2021). In these heatmaps, we represented the log_2_FC values of the genes based on the differential expression previously performed. In this way, the heatmaps allow us to cluster samples according to gene expression alteration. The measurement of dissimilarity between sets of observations was calculated using the euclidean distance. To specify the dissimilarity of the pairwise distances, the complete-linkage algorithm was used for the hierarchical clustering.

## Results

### Analysis of Plasma Membrane Fluidity of THP-1 Cells Infected With *L. infantum* Clinical Isolates

Membrane fluidity is known to affect the function of biomolecules residing within, or associated with, the membrane structure. For example, the binding of some peripheral proteins is dependent on membrane fluidity (Heimburg and Marsh, 1996). Membrane-dependent functions, such as phagocytosis and cell signaling, among others, can be regulated by the fluidity of the cell-membrane (Helmreich, 2003; Zhou et al., 2015).

Changes to plasma membrane fluidity in THP-1 cells infected with *L. infantum* lines from TF patients were evaluated by fluorescence anisotropy spectroscopy analysis using the fluorescent probes DPH and TMA-DPH. Since this parameter gives an estimation of the probe free rotation in the lipid bilayer, a higher anisotropy value should correspond to a decrease in the membrane fluidity. TMA-DPH is located near the surface while DPH is found deeper in the plasma membrane, and thus, each one reports the fluidity of a different region of the bilayer (Liang et al., 2004; Poojari et al., 2019). As expected, we obtained higher anisotropy values with TMA-DPH versus DPH probe (**Table 1**), due to the fact that the surface bilayer of plasma membrane is less fluid than the internal plasma membrane, as a consequence of lower amounts of cholesterol, as previously described (Do Canto et al., 2016). As can be seen in **Table 1**, the surface fluidity determined using TMA-DPH showed similar values in all samples, thus suggesting that there are no major changes at this level. However, measurements of THP-1 plasma membrane fractions fluidity using the DPH probe showed significant differences between the phagocytosis control and all the THP-1 cell samples infected with *L. infantum* lines from patients with TF (Hi-L2070, Hi-L2165, Hi-L2255 and Hi-L2221; **Table 1**). Indeed, the plasma membrane of these THP-1 infected cells was less fluid than in the controls (phagocytosis control and untreated cells). Additionally, we found significant differences in DPH anisotropy between THP-1 cells infected with *L. infantum* from TF isolates versus THP-1 cells infected with the reference *L. infantum* line (Hi-LJPC) (**Table 1**).

**Table 1:** Fluorescence anisotropy data for THP-1 cells.

	rDPH	rTMA-DPH
Uninfected cells	0.170 ± 0.008	0.251 ± 0.008
Phagocytosis control	0.159 ± 0.005	0.261 ± 0.008
Hi-LJPC	0.159 ± 0.004	0.271 ± 0.004
Hi-L2070	0.179 ± 0.003 *Ψ	0.271 ± 0.006
Hi-L2165	0.190 ± 0.003 *Ψ	0.266 ± 0.013
Hi-L2255	0.208 ± 0.001 *Ψ	0.273 ± 0.008
Hi-L2221	0.179 ± 0.004 *Ψ	0.268 ± 0.009

rDPH and rTMA-DPH indicate the steady-state fluorescence anisotropy values of the probes DPH and TMA-DPH incorporated in plasma membrane fractions of THP-1 cells. The assays were measured at 37°C and represent the mean ± standard deviation for at least three independent assays. Significant differences were determined using Student’s t-test (*p < 0.05 vs Phagocytosis control; ^Ψ^p < 0.05 vs Hi-LJPC).

Overall, this study showed that these *Leishmania* clinical isolates from patients with leishmaniasis and TF are able to modulate plasma membrane fluidity in infected host cells, which could partially explain the parasite’s ability to survive in the host macrophages, thereby contributing to TF.

### Determination of Cholesterol Content in Plasma Membrane Fractions of Infected THP-1 Host Cells

Several factors could affect the fluidity of plasma membranes: (i) the length and the degree of saturation of the fatty acids that compose the lipid bilayer, (ii) temperature, and (iii) the cholesterol content of the membranes, among others (Gennis, 1989).

**Figure 1:**
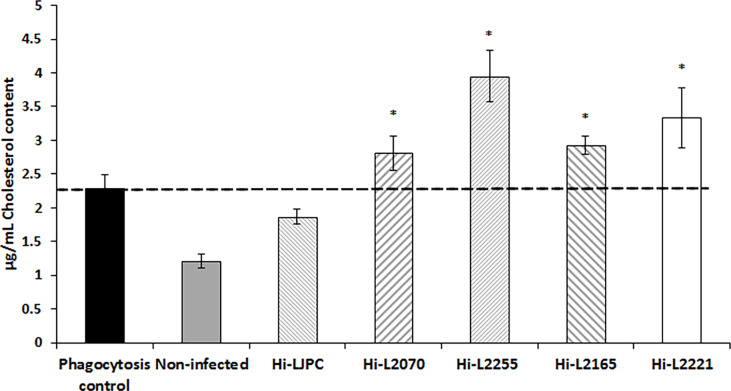
Cholesterol content in plasma membrane fractions of THP-1 cells infected with clinical TF *L. infantum* lines. The cholesterol content of the plasma membrane fractions isolated from THP-1 cells was measured using the Amplex Red Cholesterol kit, as described in the Materials and Methods section. The dashed line indicates the fluorescence value for the control. Values are the mean ± SD of three independent experiments. Significant differences versus the phagocytosis control were determined using Student’s *t*-test (*p < 0.05).

Considering that our data showed a significant decrease in the fluidity of the plasma membranes of *Leishmania*-infected THP-1 cells, we decided to analyze the cholesterol content in these samples, using the Amplex Red Cholesterol Assay kit, which is based on the oxidation of cholesterol and generation of hydrogen peroxide, with subsequent oxidation of the Amplex red reagent to produce a fluorescent compound. The results showed a significant increase in the cholesterol content of the plasma membrane fractions of THP-1 cells infected with TF *Leishmania* lines in comparison with the control (heat-killed phagocytosed parasites; **Figure 1**). The biggest difference was found for Hi-L2255, which generated an increase in the cholesterol concentration to 4 µg/mL. In contrast, THP-1 cells infected with the reference control line (Hi-LJPC), and non-infected cells, exhibited a significant decrease in cholesterol content (**Figure 1**). These results are consistent with our data for plasma membrane fluidity obtained for the different *Leishmania*-infected host cells (**Table 1**), as an increase in the cholesterol content of the plasma membrane fractions should lead to a decrease in the fluidity of the plasma membranes mainly affecting the hydrophobic core of the bilayer, as previously described (Gennis, 1989; Shrivastava et al., 2008).

Additionally, our results indicate that the infection of host THP-1 cells with heat-killed or TF parasites seems to increase the cholesterol content of plasma membrane fractions compared with uninfected cells, which showed the lowest cholesterol values (**Figure 1**).

### Cholesterol-Related Enriched Routes (GO terms)

After analyzing the results obtained for plasma membrane fluidity and cholesterol content in membranes from THP-1 cells infected with the different *Leishmania* lines, we examined the cholesterol-related enriched pathways (GO: BP terms) by analyzing the RNA-seq data for the infected host cell lines used in this study (described in the Materials and Methods section).

**Figure 2:**
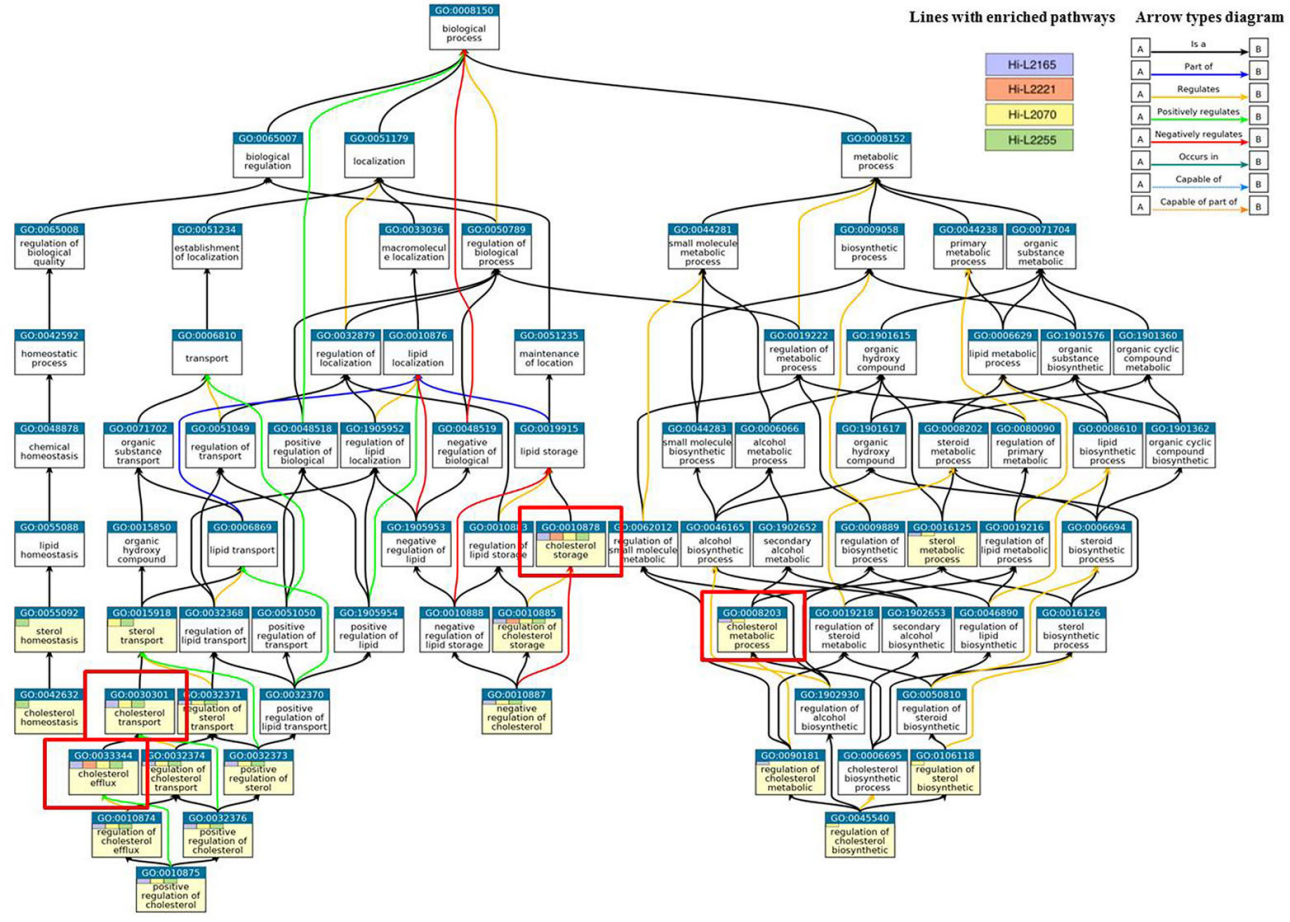
Gene Ontology (GO) enrichment analysis of the cholesterol pathway in the biological process category. Enriched pathways of THP-1 cells infected with the different *Leishmania* lines are highlighted in yellow. Cell lines with enriched routes are indicated by colored boxes (blue, Hi-L2165; red, Hi-L2221; yellow, Hi-L2070; and green, Hi-L2255). The most relevant pathways for the study are framed with a red square. The color of arrows indicates the effect of pathways. Snapshot can be downloaded from ebi.ac.uk/QuickGO.

These enriched terms are highlighted with a yellow background in **Figure 2**, and the THP-1 cells infected with different *Leishmania* lines from TF in which they appear enriched are indicated by colored boxes. In addition, all the pathways related to the enriched terms are also shown (**Figure 2**). These GO terms are shown in an ancestor chart from the most general “biological process” (top of **Figure 2**), to the more specific sterol-related term (bottom of **Figure 2**). Thus, three main differentiated terms that are related to each other can be observed: (i) “metabolic process”, (ii) “biological regulation”, and (iii) “localization” (**Figure 2**).

According to previous studies, the increase in cholesterol in the plasma membrane fractions of cells infected with TF lines might be caused mainly by changes in storage, synthesis, and transport (Samanta et al., 2017). Consequently, we focused our analysis on genes belonging to the following enriched pathways: (i) “cholesterol storage” included in “localization”; (ii) “cholesterol metabolic process” directly related to the “metabolic process” category, and finally encompassed into “biological regulation”; (iii) “cholesterol transport”; and (iv) “cholesterol efflux”.

### Heatmap and Overview of the Most Relevant Genes in Cholesterol-Related Enriched Routes for *Leishmania*-Infected THP-1 Cells

**Figure 3:**
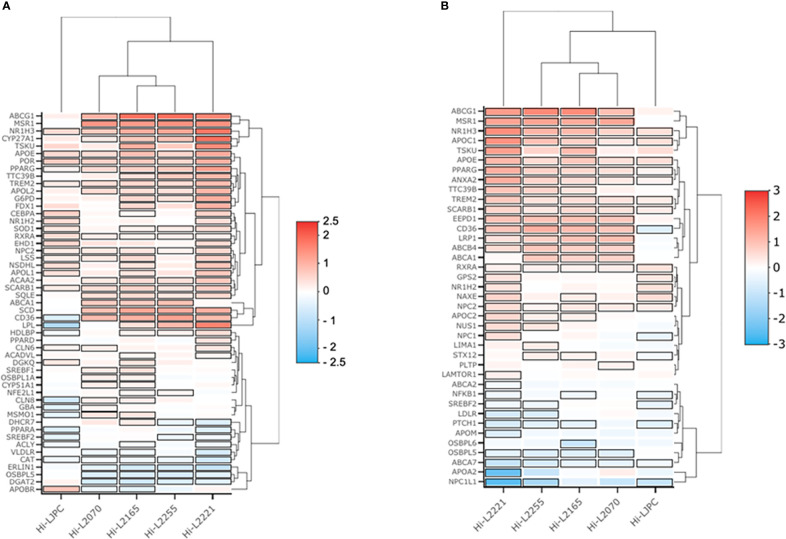
Heatmaps of genes from four selected cholesterol GO biological processes. The heatmap depicts the expression profile of genes from THP-1 cells infected with *L. infantum* lines. Red represents upregulation and blue downregulation. **(A)** Log_2_FC values for genes of the enriched pathways cholesterol storage (GO:0010878) and cholesterol metabolic process (GO:0008203). **(B)** Log_2_FC values from genes of the enriched pathways cholesterol efflux (GO:0033344) and cholesterol transport (GO:0030301).

In order to represent the differences between genes integrated in the enriched GO terms for *Leishmania*-infected THP-1 cells, two heatmap plots were generated. One of these plots comprised the genes belonging to “cholesterol metabolic process” and “cholesterol storage” (**Figure 3A**), and the other grouped the genes related to “cholesterol transport” and “cholesterol efflux” (**Figure 3B**). Both heatmaps provided an overview of the distribution of the genes upregulated and downregulated in the THP-1 cells infected with the different *Leishmania* lines. As shown in **Figure 3**, the Hi-L2221 line exhibited the greatest differences in terms of gene expression (high positive or low negative log_2_FC values), whereas Hi-L2255, Hi-L2165 and Hi-L2070 were similar to each other. Hi-LJPC, in turn, presented medium log_2_FC values for the vast majority of genes.

Amongst the biological processes studied, we highlighted some DEGs as key factors that could explain the high cholesterol present in the plasma membrane fractions of THP-1 cells infected with *L. infantum* lines from TF. Thus, *NR1H3*, which codes for an important transcription factor (liver X receptor, LXR) was overexpressed in all lines except Hi-LJPC. LXR regulates the uptake and efflux of cholesterol by acting directly on *ABCG1* and *ABCA1* (Yang et al., 2006); these genes were found to be upregulated in Hi-L2165, Hi-L2070 and Hi-L2255. *APOE* is also regulated by LXR, and was found to be one of the DEGs in Hi-L2165 and Hi-L2221. In the “cholesterol metabolic process” pathways, *SCD*, which codes for a desaturase involved in biosynthesis, was overexpressed in cells infected with all different *Leishmania* lines from TF. *CYP27A1*, *LPL* and *MSR1* were some of the relevant DEGs found in some of the lines and included in the GO category “cholesterol storage”. Finally, we observed that the genes *APOC1*, *LRP1*, *SCARB1*, *CD36*, *VLDLR*, *LDLR*, *APOA2*, *APOC2* and *ABCB4*, which were grouped in the categories related to cholesterol efflux, uptake and transport, were differentially expressed in the lines mentioned in **Table 2**.

**Table 2:** Most relevant DEGs of cholesterol routes in THP-1 cells after infection with *L. infantum* lines.

Gene ID	Function	Lines	Log_2_FC	FDR-value
*ABCG1*	Cholesterol efflux across plasma membrane	Hi-L2165 Hi-L2070 Hi-L2255	0.693 0.701 0.840	6.678E-36 1.825E-33 1.358E-30
*MSR1*	Endocytosis of modified low density lipoproteins (LDLs)	Hi-L2165 Hi-L2221 Hi-L2070 Hi-L2255	1.348 1.452 1.401 1.451	3.822E-115 4.852E-94 1.658E-103 3.711E-50
*NR1H3*	Control the expression of genes important for cholesterol uptake, efflux, transport, and excretion in multiple tissues	Hi-L2165 Hi-L2221 Hi-L2070 Hi-L2255	1.104 1.788 0.715 1.182	7.752E-23 4.469E-80 7.598E-19 4.752E-30
*CYP27A1*	Cholesterol homeostasis	Hi-L2165 Hi-L2221 Hi-L2255	0.764 1.868 0.836	3.743E-16 1.448E-50 8.142E-18
*APOC1*	Central role in high density lipoprotein (HDL) and very low density lipoprotein (VLDL) metabolism	Hi-L2165 Hi-L2221 Hi-L2255	0.751 1.731 0.986	3.200E-26 2.884E-101 3.829E-35
*APOE*	Essential for the normal catabolism of triglyceride-rich lipoprotein constituents	Hi-L2165 Hi-L2221	0.872 1.127	3.738E-41 9.949E-88
*LRP1*	Low-density lipoprotein receptor	Hi-L2165 Hi-L2221 Hi-L2070 Hi-L2255	0.972 0.478 1.032 0.748	2.457E-34 1.737E-15 1.452E-83 7.301E-36
*SCARB1*	Receptor for high density lipoprotein cholesterol (HDL)	Hi-L2165 Hi-L2221	0.608 0.684	1.191E-15 1.021E-21
*ABCA1*	Remove cholesterol from the endosomal/lysosomal compartment	Hi-L2165 Hi-L2070 Hi-L2255	0.694 0.701 0.841	6.679E-36 1.825E-33 1.358E-30
*SCD*	Desaturase involved in the biosynthesis of cholesterol	Hi-L2165 Hi-L2221 Hi-L2070 Hi-L2255	1.187 1.063 0.856 1.113	6.920E-174 1.363E-125 2.024E-62 1.278E-60
*CD36*	Binding of long chain fatty acids and may function in the transport and/or as a regulator of fatty acid transport	Hi-L2165 Hi-L2221 Hi-L2070 Hi-L2255 Hi-LJPC	1.069 0.927 0.917 1.246 -0.564	1.074E-170 5.950E-63 7.216E-133 2.297E-44 3.741E-19
*LPL*	Lipoprotein lipase involved in uptake	Hi-L2221 Hi-L2255 Hi-LJPC	1.634 0.936 -1.135	6.172E-26 2.323E-13 4.317E-09
*VLDLR*	This gene encodes a lipoprotein receptor that is a member of the LDLR family and plays important roles in VLDL-triglyceride metabolism and the signaling pathway	Hi-L2221	-0.620	2.261E-17
*LDLR*	Cell surface receptor involved in endocytosis of specific ligands	Hi-L2221 Hi-L2255	-0.748 -0.649	1.567E-06 0.0001
*APOA2*	Stabilize HDL (high density lipoprotein) structure by its association with lipids, and affect the HDL metabolism	Hi-L2221 Hi-L2255	-2.370 *-0.977*	0.010 *0.257*
*APOC2*	Plays an important role in lipoprotein metabolism as an activator of lipoprotein lipase	Hi-L2221	0.630	1.409E-13
*ABCB4*	Phospholipid efflux translocator	Hi-L2165 Hi-L2221 Hi-L2070 Hi-L2255	0.741 0.580 0.693 0.883	4.789E-05 0.0004 1.684E-06 2.655E-11

Profile of DEGs for THP-1 cells after infection with different L. infantum lines, as described in the Materials and Methods section. The analysis was based on log_2_FC and false discovery rates (FDRs). Non-statistically significant values (log_2_FC) with similar trends are shown in italics.

## Discussion

In this study, we have shown that infection with *L. infantum* lines isolated from patients with leishmaniasis who had developed TF leads to a decrease in the fluidity of the plasma membrane of the host cell together with an increase in the cholesterol content of these plasma membranes. Some authors have reported that *Leishmania* infection promotes a reduction in the plasma membrane cholesterol content in the macrophage as a result of lipid raft disruption (Sen et al., 2011). However, the changes in cholesterol levels following internalization of *Leishmania* into the macrophage remain controversial. Indeed, according to other studies, there is a relationship between *Leishmania* infection and an increase in the cholesterol levels in host cells (Osorio y Fortéa et al., 2009).

Interestingly, the cholesterol levels in uninfected cells were the lowest in this study, and were significantly higher for the heat-killed parasite phagocytosis line. It has been reported that the presence of cholesterol in the plasma membrane of macrophages is required for the effective attachment and subsequent entry of *Leishmania* (Pucadyil et al., 2004). Initially, the amount of cholesterol in the plasma membrane seems to decrease after infection (Rub et al., 2009). However, our studies were performed at a later stage of infection and, taking into account that cholesterol in the host membranes is required for entry of the parasites, the levels should rise sufficiently to allow progression of the infection. Indeed, our results suggest that phagocytosis of parasites itself induces an increase in the plasma membrane cholesterol levels for the host cells, which is reported for other microorganisms such as *Mycobacterium smegmatis* (Viswanathan et al., 2015).

Eukaryotic cells closely regulate cholesterol levels by balancing metabolism, uptake, efflux, and storage. *De novo* biosynthesis occurs with the conversion of HMG-CoA to mevalonate by HMG-CoA reductase (HMGR) or hydrolysis of cholesteryl esters primarily taken up by low density lipoprotein receptor (LDLR) (Simons and Ikonen, 2000). Both biosynthesis and uptake are regulated at the expression level by sterol regulatory element binding protein (SREBP) (Brown and Goldstein, 1997) or liver X receptor (LXR) transcription factors, which increase the transcription of HMGR and LDLR under low cholesterol conditions (Mukherjee et al., 2014; Samanta et al., 2017).

After analysis of the genes belonging to the different cholesterol-related GO biological processes mentioned, in the category related to transport (uptake and efflux) we found several genes, namely *NR1H3*, *ABCA1*, *ABCG1* and *APOE*, that are overexpressed in most of the infected cell lines, with the exception of Hi-LJPC (reference control line).

**Figure 4:**
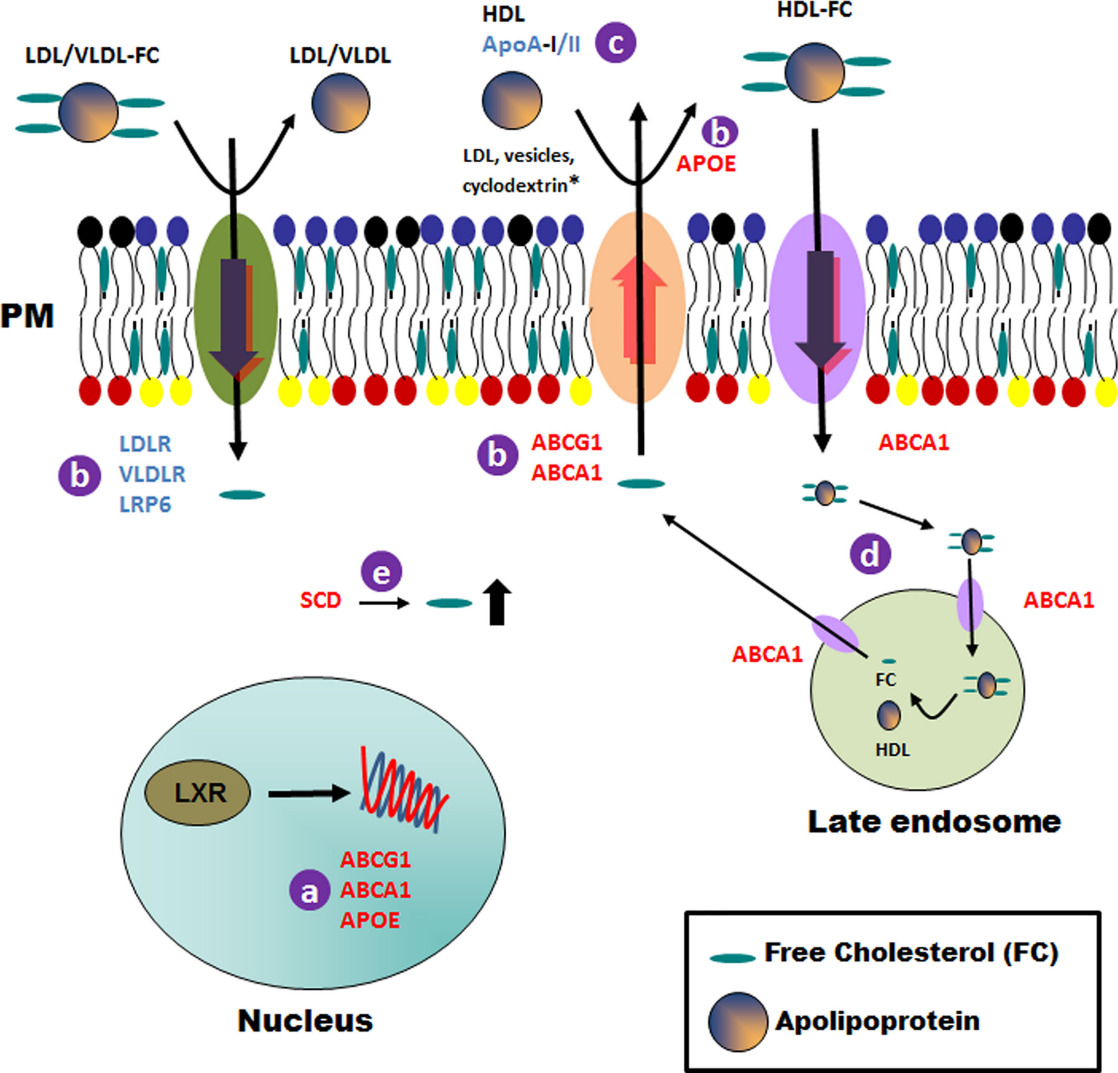
Schematic representation of general cholesterol pathways modulated in THP-1 host cells after infection with *Leishmania* lines from TF. Details of the processes that could be affected in the host cell after infection with TF *Leishmania* lines are indicated with a letter **(A–E)** and described in the Discussion section. Upregulated and downregulated genes are shown in red and blue, respectively. * For the ABCG1 transporter only. PM, plasma membrane.

The liver X receptors LXRα (*NR1H3*) and LXRβ (*NR1H2*), are members of the nuclear hormone receptor superfamily of ligand-activated transcription factors (Schulman, 2017). Previous studies identified the genes encoding the ATP-binding cassette transporters *ABCA1* and *ABCG1*, as well as the gene encoding apolipoprotein E (*APOE*), as direct *LXR* target genes (**Figure 4A**) (Yang et al., 2006). In response to increasing cholesterol content, LXR promotes the movement of cholesterol out of cells to high-density lipoprotein particles (HDL) (Zelcer et al., 2009) (**Figure 4B**). The ATP-binding cassette transporters ABCA1 and ABCG1 are mainly responsible for macrophage cholesterol efflux to the serum or HDL (**Figure 4C**) (Yvan-Charvet et al., 2010). As a result, two distinct mechanisms have been proposed to explain ABCA1-mediated cholesterol efflux from macrophages to HDL particles: (i) ABCA1-mediated cholesterol efflux to HDL can occur at the plasma membrane (**Figure 4C**) and (ii) internalization of apolipoprotein-lipid complexes to internal compartments and late secretion by ABCA1 (**Figure 4D**) (Wang et al., 2000). On the other hand, ABCG1 transporter promotes efflux of cholesterol to a variety of acceptors, including HDL, LDL and phospholipid vesicles (**Figure 4C**) (Wang et al., 2008). Finally, APOE facilitates the transfer of intracellular cholesterol to HDL particles (Zanotti et al., 2011) (**Figure 4B**).

HDL-mediated cholesterol efflux *via* LXR-regulated ABC transporters plays a key role in modulating lymphocyte proliferation and activation, and could be one of the purposes for the modulation of this route in macrophages infected with *Leishmania* lines from TF (Yvan-Charvet et al., 2010).

Apart from the transport pathways, we found that the gene *SCD* was overexpressed in all *Leishmania*-infected cell lines. This gene codes for the rate-limiting enzyme stearoyl-CoA desaturase, which is involved in fatty acid biosynthesis, primarily the synthesis of oleic acid (Wang et al., 2005). The high cholesterol content found in the plasma membranes of cells described in the manuscript could be due to an increase in cholesterol content promoted by SCD (**Figure 4E**).

Our analysis also showed a reduced expression of *APOA2*. Apolipoprotein A-II (ApoA-II) is the second most abundant protein component of HDL and is closely associated with modulation of HDL metabolism and alteration of HDL conformation by interacting with ApoA-I and other apolipoproteins (Yang et al., 2018). *APOA2* over-expression has been shown to increase the HDL-cholesterol level in animal models (Bandarian et al., 2016). Additionally, other lipoprotein receptors, such as *VLDLR*, *LDLR* and *LRP6*, amongst others, were found to be underexpressed in the present study. Briefly, a reduction in the expression of these genes could affect the transfer of cholesterol from the plasma membrane to the apolipoproteins (HDL, LDL, VLDL, etc.; **Figure 4B**) and, consequently, will result in a significant increase in the cholesterol content of the plasma membrane, as observed in our experimental studies.

To summarize, we suggest that the high levels of cholesterol found in the plasma membrane fractions of cells infected with the different *Leishmania* lines could be due to a combination of an increase in cholesterol biosynthesis, transport to the plasma membrane and a defect in the transfer of cholesterol from the plasma membrane to the apolipoprotein particles (**Figure 4**).

As is also the case for other intracellular pathogens, *Leishmania* parasites have developed strategies to evade host immune mechanisms in order to survive within the host. Thus, infection with *Leishmania* modifies plasma membrane cholesterol levels, thereby altering the immune response required to control the progression and evolution of this parasite infection and contributing to immune evasion of the parasites (Rub et al., 2009; Roy et al., 2016). Indeed, an excess of cholesterol in the plasma membranes of immune cells can trigger the production of autoantibodies and autoreactive T cells, thus leading to an autoimmune disease (Widenmaier and Hotamışlıgil, 2016). In this way, an increase of cholesterol in the plasma membranes of macrophages after *Leishmania* infection could generate an ineffective immune response that may contribute to TF.

Taken together, our findings underline a plausible tendency of clinical isolates of *Leishmania* from TF patients to modulate the gene expression of some host-cholesterol genes that lead to a signaling response, thereby modifying the immune response required to control the progression and evolution of this parasite infection and contributing to immune evasion of the parasites and TF in patients with leishmaniasis. However, whether this observed biological response is universal requires further studies. Finally, our findings could have important implications in future studies aimed towards designing new therapeutic strategies against leishmaniasis in patients with TF, considering that the cholesterol levels in these patients at the time of drug delivery could be of relevance for the efficacy of antileishmanial drugs.

## Data Availability Statement

The original contributions presented in the study are included in the article/**Supplementary Material**. Further inquiries can be directed to the corresponding author.

## Author Contributions

Conceptualization, FG, JM, AP-M, and RG-H; methodology, JM, AP-M, RG-H, LT-C, EA-L, JP; formal analysis, JM, AP-M, RG-H, LT-C, EA-L, JP, and FG; writing-original draft preparation, JM, AP-M, RG-H, and FG; supervision, JM, AP-M, RG-H, and FG; project administration, FG; funding acquisition, FG. All authors have read and agreed to the published version of the manuscript.

## Funding

This work was supported by Grant RTI2018-097210-B-100 (FG), funded by MCIN/AEI/10.13039/501100011033 and by “ERDF A way of making Europe”.

## Conflict of Interest

The authors declare that the research was conducted in the absence of any commercial or financial relationships that could be construed as a potential conflict of interest.

## Publisher’s Note

All claims expressed in this article are solely those of the authors and do not necessarily represent those of their affiliated organizations, or those of the publisher, the editors and the reviewers. Any product that may be evaluated in this article, or claim that may be made by its manufacturer, is not guaranteed or endorsed by the publisher.
