# Supplementary material for: Modulation of Cholesterol Pathways in Human Macrophages Infected by Clinical Isolates of Leishmania infantum
Source: Front Cell Infect Microbiol. 2022 Apr 29;12:878711. doi: 10.3389/fcimb.2022.878711 (PMC9106381; doi:10.3389/fcimb.2022.878711)
Supplement: Supplementary file 1 [file DataSheet_1.pdf]

Supplementary Table 1. Infection parameters of THP-1 cells infected with different *Leishmania infantum* lines

Leishmania infantum line	Infection (%)	Amastigotes/macrophage (nº)
JPCM5	79.9 ± 10.0	8.4 ± 0.6
LLM2221	60.1 ± 0.6	9.6 ± 1.2
LLM2165	71.5 ± 0.2	6.9 ± 0.9
LLM2255	69.5 ± 7.3	7.9 ± 1.6
LLM2070	62.5 ± 13.1	8.8 ± 0.9

Macrophages were infected with the different *L. infantum* lines following the protocol described in Material and Methods section. For microscopy visualization, cells were fixed for 30 min at 4°C with 2.5% paraformaldehyde in PBS and permeabilized with 0.1% Triton X-100 in PBS for 30 min. Data represents the mean ± standard deviation for at least three independent assays. No significant differences appeared between the different *Leishmania* lines.

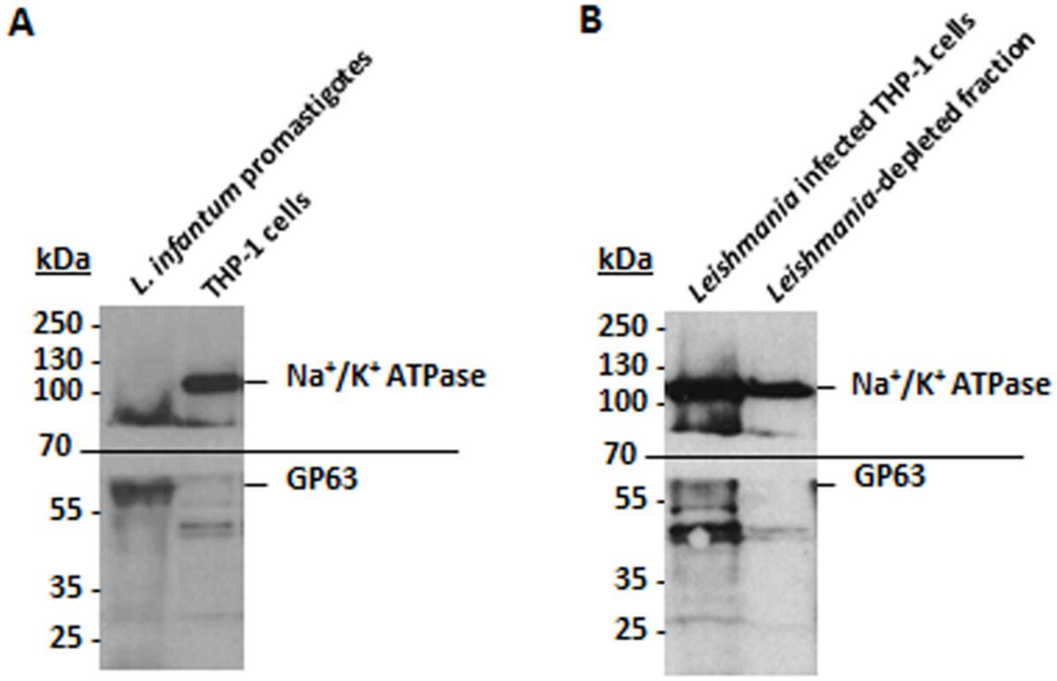

Figure S1. Western blot analysis of Na^+/K^+ ATPase and GP63. (A) *L. infantum* promastigotes of JPCM5 line and THP-1 cells lysates; and (B) protein samples (10 µg) obtained from a lysate of infected THP-1 cells (*Leishmania* infected THP-1 cells) and the fraction after *Leishmania*-depletion protocol (*Leishmania*-depleted fraction; plasma membrane) were analyzed by Western blot as described in Material and Methods. The antibodies employed were monoclonal anti-(GP63) (generated in mouse; 1:5000) or monoclonal anti-(Na^+/K^+ ATPase, α1 subunit) (generated in mouse; 1:5000).
